# Seamen's Hospital Society

**Published:** 1905-01-21

**Authors:** 


### SEAMEN'S HOSPITAL SOCIETY.

IT was stated at the quarterly Court of Governors of the Seamen's Hospital Society last week (Mr. Percival A. Nairne in the chair) that the Right Hon. Joseph Chamberlain had consented to preside at a banquet in aid of the hospital and the London School of Tropical Medicine on May 10th, and that the Duke of Marlborough had accepted the office of Chairman of the Dinner Committee. In order to bring the society into greater prominence offices have been taken at 13A Cockspur Street, where it is hoped that the models of the old *Dreadnought* which will be displayed may attract the notice of persons who may be disposed to contribute towards the care of seamen disabled in the service of the Empire, some 6,000 of whom were treated during the past three months.

